# Celery and Celeriac: A Critical View on Present and Future Breeding

**DOI:** 10.3389/fpls.2019.01699

**Published:** 2020-01-22

**Authors:** Silvia Bruznican, Hervé De Clercq, Tom Eeckhaut, Johan Van Huylenbroeck, Danny Geelen

**Affiliations:** ^1^ Plant Sciences Unit, Flanders Research Institute for Agriculture, Fisheries and Food (ILVO), Melle, Belgium; ^2^ Department of Plant Production, Ghent University, Ghent, Belgium

**Keywords:** *Apium*, hybrids, diseases, stress, nutraceuticals, petiole, hypocotyl, genetics

## Abstract

Cultivated for the crispy petioles and round, fleshy, and flavored hypocotyl celery and celeriac have over two centuries of breeding history in Europe. In this review paper we summarized the most recent advances touching when necessary the historical context of celery and celeriac breeding. In the post genomic era of research, the genome sequence of celery is only partially available. We comprised however in this paper the most important aspects of celery genetics that are available today and have applicability in celery modern cultivars development. We discussed the problems and traits that drive the main celery and celeriac breeding goals, like hybrid seed production, disease resistance, and interesting enlarged hypocotyl and petiole characteristics. Besides the classical breeding traits we covered the potential of integration of existing cultivars as sources for consumer oriented traits like nutraceuticals and health promoting substances. Sustainability is a subject that is continuously growing in popularity and we looked at the genetic base of celery and celeriac that makes them sources for abiotic stress resistance and candidates for phytoremediation. We explored the fundamental concepts gained in various fields of celery and related species research, as resources for future improvement of celery and celeriac germplasm. We forecast what the next years will bring to *Apium* breeding.

## Introduction

**Figure 1:**
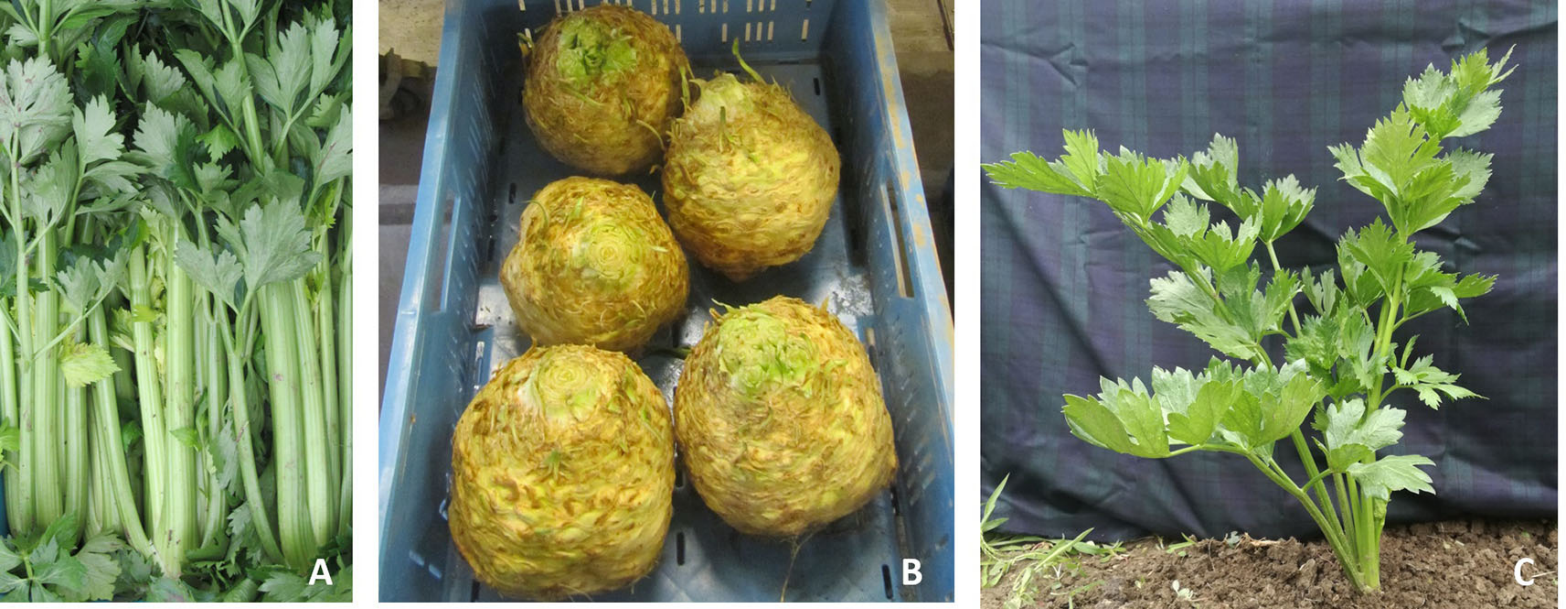
*Apium graveolens* botanical varieties: *dulce* **(A)**, *rapaceum* **(B)**, and *secalinum* **(C)**.

Celery and celeriac, *Apium graveolens* (2n = 2x = 22) are worldwide cultivated vegetables and aromatic plants with important pharmaceutical properties. *A. graveolens* includes three botanical varieties: var. *rapaceum* known as celeriac, cultivated for its edible hypocotyl, which is popular in Europe, var. *secalinum*, for smallage production mostly cultivated in Asia and var. *dulce*, popular for its crisp stalks in America and Western Europe (**Figure 1**). Celery originates from the Mediterranean basin and breeding started in the 19th century in Italy when the cultivars separated in the white, ‘Golden Self-blanching’, and green, ‘Pascal’ (Quiros, 1993a).

During 2017, 79,404 t of celery were produced in the USA (AgMRC, 2018) while the European Union produced 335,990 t of celery and 526,000 t of celeriac (Eurostat, 2019).

The breeding goals of modern celery and celeriac cultivars are established by the local markets. The USA market is demanding resistance to biotic stress especially *Fusarium* yellow. Within Europe the common goals are high uniformity and yield, whereas bolting resistance, interesting petioles, and enlarged hypocotyl characteristics are regional breeding goals. The most notable reviews that address celery breeding are the ones of Quiros (1993a), which is over 25 years old, and Li et al. (2017), which is a general review and does not focus specifically on breeding. Practical celery and celeriac breeding is done by the private sector that keeps many key steps confidential. On the contrary, academic research focuses on unraveling fundamental insights. It is therefore cumbersome for as well breeders, academics, and other stakeholders to obtain a complete overview of current celery breeding and to foresee future breeding goals. We seek to address these issues by reviewing all the key goals of *Apium* breeding and enumerating the scientific literature that may contribute to current breeding targets or could be a stepping stone for innovation and future developments.

In the following paragraphs we will discuss achievements in celery and celeriac breeding and research. We tackle scientific literature of worldwide importance, while focusing on breeding targets from a European perspective. The last section of the review is a discussion in which we speculate on the future of *Apium* breeding, based on scientific knowledge and company insights.

## Celery and Celeriac Breeding

### Hybrid Breeding and Male Sterility

Hybrid breeding remains one of the major goals of celery and celeriac breeding in Europe. With high uniformity, increased yield, vigor, and disease resistance, heterosis allures growers and breeders equally. Celery and celeriac breeding is done mainly *via* open-pollination and this results in high plant to plant variation which has a detrimental economic impact (Rubatzky et al., 1999). Hybrid cultivars are available on the market in Europe and USA, but still in a small number when compared to open pollinated (OP) varieties. The difficulty of obtaining celery and celeriac F1 hybrid seeds is imposed by the lack of emasculation means for umbelliferous flowers. *Apium* flowers, tiny and organized in inflorescences called umbels, are highly prone to self-pollination (Pierce and Duda, 2011). The most efficient emasculation method is the use of totally male sterile lines. Male sterility, characterized by the inability of the plant to produce fertile pollen, can be genetically or cytoplasmically determined (Colombo and Galmarini, 2017). Genetic male sterility segregates and therefore these lines are vegetatively propagated in breeding programs. Conversely, cytoplasmic male sterility (CMS) and cytoplasmic genic male sterility (CGMS) are determined by mitochondrial encoded genes, hence are maternally inherited and are economically advantageous. The first celery male sterile line, the Iranian accession ‘P1229526’, described by Quiros et al. (1986), was determined by a single recessive gene, *ms-1*. Gao et al. (2006) identified a male sterile plant, ‘01-3A’ in a commercial field trial of the inbred line ‘01-3’. They concluded that the male sterility was controlled by two nuclear recessive genes (Gao et al., 2009) and they used this line to create the celery hybrid ‘Jinqi 2’ (Gao et al., 2008). Reports on CMS sources in *Apium* are elusive. In 1993, Dawson stated in the “Grower” magazine that he has introgressed CMS in celery from an unidentified wild celery plant, albeit unstable. Some of today’s hybrid celery and celeriac cultivars were obtained using CMS but the mechanisms and origin are contained by the breeding companies.

However, CMS has been described in other Apiaceae species. These systems could be transferred in celery and celeriac by asymmetric protoplast fusion. An efficient protocol for protoplast regeneration, a first step in the process, was recently published by Bruznican et al. (2017).

### Breeding for Disease Resistance

Late blight is the major celery and celeriac leaf disease caused by *Septoria apiicola* which is totally detrimental under poor crop management practices (Raid, 2004).

Natural resistance from *Petroselinum hortense* (parsley) (Honma and Lacy, 1980) was introgressed in celery. However the progenies of the crossings between celery and parsley were susceptible to *Septoria* in another study (Edwards et al., 1997). It was suggested that with each successive backcrossing, the resistance to *Septoria* was bred out. *Septoria* resistance was identified in the wild species *Apium nodiflorum*, *Apium chilense*, and *Apium panul*; the latter two were crossed to celery (Ochoa and Quiros, 1989). The F1 hybrids resulting from these crosses were slightly less resistant than their resistant parent, indicating incomplete dominance of the resistance trait.

*Fusarium oxysporum*, a soil-borne fungus, causes *Fusarium* yellow disease in celery and celeriac for which there are no effective treatments (Raid, 2004). In the mid-80’s a *Fusarium* resistant celeriac landrace was crossed with the susceptible celery cultivar ‘Tall Utah 52-70R’. The resistance trait was determined by one dominant gene and other independent quantitative genes (Orton et al., 1984a). This resistance was used to develop the resistant line ‘UC1’ (Orton et al., 1984b) which was backcrossed in elite varieties in the early-90’s. The resulting resistant lines, UC8-1, UC10-1, and UC26-1 were used for development of commercial cultivars (Quiros et al., 1993). Other *Fusarium* resistant celery plants were obtained through somaclonal variation during regeneration from cell suspensions by selection (Toth and Lacy, 1991). After five generations of self-pollination combined with *Fusarium* resistance selection, a stable resistant line, MSU-SHK5 was created (Lacy et al., 1996) but no follow-up of its use in commercial cultivars is available.

In 2013, a new race, *F. oxysporum* f. sp. *apii* race 4, was identified in California (USA) and none of the commercial cultivars available display resistance to it (Epstein et al., 2017). Research at the University of California, Davis, focused on searching resistance sources to this new race. One hundred twenty celery, 66 celeriac, 25 smallage, 5 celery x celeriac, and 15 miscellaneous *Apium* species were screened. A celery from China and three potentially resistant celeriac accessions from Turkey were identified (Epstein, 2017).

Early blight, caused by the fungus *Cercospora apii*, is a highly transmissible *Apium* disease that requires an intensive and expensive disease control management. Early work by Wolf and Scully in the 90s resulted in a series of cultivars for the Florida region. ‘Floribelle M9’ displayed at the moment of its creation superior resistance to early blight compared to other resistant cultivars (Wolf and Scully, 1992). Its resistance to *Cercospora* can be traced to a Turkish accession of celeriac. ‘Floribelle M9’ was further used for development of the early blight resistant cultivar ‘FBL 5-2M’ (Scully et al., 1995).

Celery mosaic virus (CeMV) is the most common viral disease of celery and is transmitted by aphids (Raid, 2004). D’Antonio et al. (2001) identified a single recessive locus for resistance to CeMV. Ruiz et al. (2001) found markers linked to this *cmv* gene. These markers can facilitate introgression and selection of the virus resistance in other celery varieties. McCormick attempted to produce celery and carrot plants resistant to CeMV and carrot virus Y (CarVY) using post-transcriptional gene silencing technology. They were unsuccessful in obtaining celery resistant plants, presumably due to the recalcitrance of the used celery cultivars toward *Agrobacterium*-mediated transformation (McCormick, 2006).

### Breeding for Insect Pest Resistance

Beet armyworm, *Spodoptera exigua*, is a celery and celeriac pest difficult to control chemically. Resistance against this insect was identified in *Apium prostratum* (Diawara et al., 1992). Moreover, celery somaclonal lines resistant to *Fusarium* yellow display a significant increase in beet armyworm resistance (Diawara et al., 1996). Meade and Hare (1991) compared 13 cultivars of var. *rapaceum*, *dulce*, and *secalinum* for resistance against the beet armyworm and observed that the resistance of ‘Kockanski’ cultivar against *S. exigua* was correlated with an increase in the content of a sedanolide (3-n-butyl-4,5-dihydro-isobenzofuranone) (Meade et al., 1994).

The leafminer *Liriomyza trifolii* causes yield loss and decreased marketability. Natural resistance to this pest was reported in *A. nodiflorum* (Trumble et al., 1990) and *A. prostratum* (Trumble and Quiros, 1988). Trumble et al. (2000) crossed celery with *A. prostratum* and produced a series of backcrossings to celery. The F1 hybrid and some of the backcrossed lines maintained the resistance to the leafminer as observed in the wild parental accession.

*Apium* is susceptible to infestation by 13 different parasitic nematodes species (Sikora and Fernandez, 2005). The most infectious nematodes belong to the genus *Meloidogyne*, with *Meloidogyne javanica* and *Meloidogyne incognita* being common in tropical regions while *Meloidogyne hapla* is a common species in temperate and cooler regions (Sikora and Fernandez, 2005). The difficulty of controlling these parasitic infestations is associated with the large number of plant species that serve as hosts for *Meloidogyne* nematodes.

The northern root-knot nematode, *M. hapla* can cause up to 5% losses in celery cultivation (Melakeberhan and Wang, 2012). This pest is prevalent in other vegetable crops as well and its control with broad spectrum pesticides is restricted. Melakeberhan and Wang (2012) tested five celery cultivars for resistance to *M. hapla* and concluded that two of them, ‘Green Boy’ and ‘Dutchess’, developed significantly lower numbers of adult nematodes.

A putative source of nematode resistance genes may be found in the related Apiaceae vegetables. Carrot resistance toward *M. hapla* has been well investigated and resistant lines were identified (Wang and Gabelman, 1993). The resistance is determined by two different homozygous recessive genes (Wang and Goldman, 1996).

*M. incognita* and *M. javanica* infestation can occur in celery, although not often (Vovlas et al., 2008). Yet no sources or mechanisms of resistance were described. In carrot, resistance to these nematodes is determined by one or two duplicated, linked genes, designated locus *Mj-1* (Simon et al., 2000). A second resistance-to*-M. javanica-*region, *Mj-2*, was found in the carrot breeding material derived from Asian germplasm (Ali et al., 2014). These resistance sources available in carrot germplasm along with the identified resistance-associated markers could serve as valuable breeding materials and tools for resistance introgression in *Apium*.

*Meloidogyne chitwoodi* is an aggressive nematode, which is under the quarantine status in Europe (Zijlstra, 2008). It can affect celeriac production by penetrating the enlarged hypocotyl and producing skin abnormalities and browning under the skin. An evaluation of the resistance status of several cultivars is currently ongoing at Flanders Research Institute for Agriculture, Fisheries and Food (ILVO) (Belgium).

### Breeding for Late Bolting

Celery and celeriac require an extended cold period in order to flower. During the first year of development, bolting starts as the main stem begins to form a floral stalk. Premature bolting may happen because of stress induced suppression of vegetative growth and this has a major impact on the yield and quality of celery because the inflorescence stalk drains the energy away from the leaves and petioles (Quiros, 1993a).

The dependence on a cold period is highly variable in *Apium* and is genetically determined [reviewed by Quiros (1993a) and Li et al. (2017)]. In general, annual celery cultivars bolt easily while biennial cultivars display weak to strong bolting resistance. The crossings performed by Bouwkamp and Honma (1970) represent the starting point of early bolting resistance breeding in celery.

Although most of the European celery and celeriac cultivars are nowadays biennial and sufficiently resistant to bolting in the first growing season, there are still regions where growers are confronted with early bolting. In Southern Europe (Spain) the plants sown in autumn have good overwinter period and are stimulated to flower in the first year.

A deeper understanding of *Apium* bolting has been gained through transcriptomic data that identified orthologues of the flowering genes *ELF7 and ELF8* (*EARLY FLOWERING 7 and 8*), *FCA* (*FLOWERING TIME CONTROL*), *FLC* (*FLOWERING LOCUS C*), *RKF3* (*RECEPTOR-LIKE KINASE IN FLOWERS 3*), *TM6* (*TOMATO MADS-BOX GENE 6*), *AP3* (*APETALA 3*), *TFL2* (*TERMINAL FLOWER 2*), and *PIE1* (*PHOTOPERIOD-INDEPENDENT EARLY FLOWERING 1*) (Fu et al., 2013). Allelic variations of these genes are potentially important for the late bolting trait and could be used in the future for molecular marker assisted selection and enable target development for the new emerging breeding techniques.

### Breeding for Petiole Traits

The petioles of *dulce* varieties are long with solid stems, sweet in flavor, and tender in texture, while the petioles of varieties *secalinum* and *rapaceum* are shorter and hollow, bitter in flavor, and fibrous in texture (Honma and Lacy, 1980; Quiros, 1993a; Rubatzky and Yamaguchi, 1997). Throughout time many competitive petiole varieties were developed with a wide variation of color, shape, crispness, pithiness, stringiness, and flavor.

The color of the celery petiole is subjected to the consumer’s preference. It can vary from green, light green, yellow, light yellow to white. Two major groups of celery exist: the yellow/golden so called ‘self-blanching’ cultivars with almost colorless petioles and foliage, like ‘Golden Self Blanching’ and ‘Celebrity’ and the green cultivars with deep green petioles and leaves like Utah types and summer Pascal types (Malhotra, 2006; Callens and Vanheule, 2012).

Although the selection of green and yellow cultivars started more than one century ago, the genetics underlying it were unknown for a long time. Wang et al. (2010) observed in celery line ‘CE188H’ that the completely yellow phenotype is under the control of a recessive gene named *YEL*. In another study, it was determined that a dominant gene *WP1* controlled the white petiole trait and a Sequence Characterized Amplified Region (SCAR) marker tightly linked to the gene was identified (Han et al., 2012). Fu et al. (2013) identified the celery orthologues of eight unigenes involved in chlorophyll biosynthesis and four involved in chlorophyll catabolism that can potentially control petiole color, as the yellow petiole mutation is associated with a decrease in chlorophyll content in celery (Wang et al., 2010).

*Apium* petiole color is not limited to yellow and green, also red pigmentation has been reported in celeriac cultivars originating from Eastern Europe. The red pigmentation is caused by accumulation of anthocyanin, a secondary metabolite. The anthocyanin biosynthesis was studied in ‘Nanxuan liuhe’ purple celery variety and it was proposed that apigenin and anthocyanins share a common precursor, naringenin. Overexpression of the gene that encodes the enzyme responsible for the conversion of naringenin in apigenin, *AgFNS* (*FLAVONE SYNTHASE*), results in decreased expression levels of other genes involved in anthocyanin biosynthesis (Tan et al., 2017).

Pithiness is the tendency of celery mature petioles to develop a spongy, hollow, and white discoloration of the parenchymatous tissue. It causes yield losses when it occurs at an earlier stage. The hollowness trait is naturally present in annual *secalinum* varieties and in the Thai accession ‘A143’ it is a polygenic trait (Huestis et al., 1993). The hollow trait of the accession ‘China B’ was combined with the mild flavor of the ‘Hill’s Special’ cultivar in a new variety, ‘ADS-9’, which has a cylinder shaped petiole which can be used as a drinking straw and is considered a new class of celery, the hollow-stem celery (Pierce and Duda, 2011).

Collenchyma, a tissue with thickened cell walls, is the major mechanical support constituent of the celery petiole (Chen et al., 2017). Fu et al. (2013) identified 100 celery homologues of genes involved in the metabolism of cellulose and pectin. More detailed investigation of the role of these genes in celery could reveal whether any of them are key players in determining the hollow or solid petiole trait, so that they can be used in breeding programs.

Crispness is a trait described as a combination of light texture, a snap, clean break, a high-pitch sound during chewing, and lack of fibrousness (Fillion and Kilcast, 2002). Despite the fact that petiole crispness is a sensory trait difficult to quantify, it is highly regarded by the consumers and breeders who strive to bring interesting varieties on the market. The fibrous character of celery is influenced by external factors such as environmental conditions or date of harvest (Guerra et al., 2010), but it depends also on the intrinsic factors, such as morphology of the vascular bundles. Raffo et al. (2006) compared several celery cultivars and concluded that the green cultivars ‘Early Green’ and ‘Octavius’ were more fibrous than the yellow varieties ‘Golden Boy’ and ‘Sedano Bianco di Sperlonga’. Vina and Chaves (2003) observed a correlation between an increase in lignin content and the accentuation of tissue fibrosity in time during celery cold storage. Jia et al. (2015a) studied lignin biosynthesis in celery petiole and observed that the lignin content in the vascular bundle increases with development. The products of 11 highly conserved genes are responsible for lignin biosynthesis in celery petiole and leaf at different developmental stages. These insights in the mechanism of lignin biosynthesis can aid both celery classic breeding, by providing selection markers, and the new breeding techniques, such as clustered regularly interspaced short palindromic repeats (CRISPR)/CRISPR-associated protein 9 (Cas9), by rendering specific targets that can be modified to obtain new petioles textures.

Flavor of the celery petioles is the impression given by the release of volatile compounds during chewing (Raffo et al., 2006). The volatile compounds responsible for the specific celery flavor of leaves and petioles have been long studied and phthalides and terpenes were considered to account for the specific odor (Gold and Wilson, 1963). Uhlig et al. (1987) managed to separate *via* high-performance liquid chromatography (HPLC) the phthalides extracted from stems of celery and correlated the data with sensory evaluation of the petioles. They concluded that the concentration of phthalides varies throughout different celery cultivars and that sedanolide, a minor component of the phthalides fraction, accounts mostly for the perception of the celery flavor. The difference in flavor between celeriac and celery could be due to the fact that celery is richer in volatile compounds, containing 68 identified compounds in contrast with only 46 contained by celeriac (Macleod and Ames, 1989). Moreover, celeriac contained very low levels of caryophyllene and lacked completely β-selenine and seven other sesquiterpene hydrocarbons that are essential flavor components of celery. Van Wassenhove et al. (1990) analyzed four cultivars of blanching celery and six cultivars of celeriac and established that the concentrations of phthalides and terpenes vary within both blanching celery and celeriac cultivars. In the aforementioned study of Raffo et al. (2006) regarding sensory perception of different celery cultivars, a trained panel evaluated the flavor. The green petiole cultivar ‘Early Green’ was perceived as having a significantly stronger celery specific flavor than the other cultivars. The study concluded that the flavor of celery does not depend on the basic tastes and that there could be an association between bitterness and other sensory traits such as fibrousness (Raffo et al., 2006).

All these reports on the attempts of unraveling the celery flavor indicate that it is becoming easier for the breeders to assess the desired flavor trait during the selection process and besides using expert panels, which are the ultimate indicatives of the taste, they can use chemical methods to specifically detect flavor compounds. These insights can speed up the breeding process by modifying flavor compound biosynthetic pathways.

The length of the petiole is the distance from the base of celery petiole to the insertion of the first pair of leaflets. According to the classification used in celery breeding, petioles with the length to the joint between 20 and 30 cm are considered medium size, those exceeding 30 cm are considered long, and those under 20 cm are considered short (Deneer and Glawe, 2017). Without any knowledge on the genetics underlying the length trait, breeders performed crossings and selected plants based on this criterion for decades, developing commercial or patented cultivars of various petiole lengths. The vast majority of cultivars available today in Europe have long and super long petioles (exceeding 40 cm). The emergence of these super long petiole cultivars allowed the processing of petioles into celery sticks, a new category of celery product. The accession ‘41902’, patented by Rijk Zwaan is a very short petiole celery variety having only 9.8 cm in length, which was designed to be consumed by a single person (Deneer and Glawe, 2017).

The molecular mechanisms underlying petiole development are understudied in celery. However, the expression level of *LEP* (*LEAFY PETIOLE*) gene, involved in petiole development in *Arabidopsis* (van der Graaff et al., 2000), was described in celery cultivars ‘Ventura’ and ‘Liuhe Huangxinqin’ (Jia et al., 2015c). Petiole length is determined by the interplay between cell length and cell number (Weijschede et al., 2008). We searched in the celery transcriptome data sets available from the studies of Fu et al. (2014); Jiang et al. (2014), and Li et al. (2014a), deposited on National Center for Biotechnology Information (NCBI), for sequence similarities with *Arabidopsis* genes controlling petiole length. By performing a discontiguous megablast we found that sequences similar to *PHYB* (*PHYTOCHROME B*), which controls individual cell length, *GAI* (*GIBBERELLIC ACID INSENSITIVE*), *GA1* (*GIBBERELLIC ACID1*), *ROT3* (*ROTUNDIFOLIA3*) that are involved in cell elongation and proliferation under strong light (Tsukaya et al., 2002), and *ER* (*ERECTA*), which controls differential petiole elongation (van Zanten et al., 2010), are somewhat conserved in celery as well. The presence of the true orthologues of these genes in celery still needs to be checked and their function must be studied in order to be able to apply them in celery breeding programs.

In the production of celery stalks, the leaf upper parts represent 20 to 30% of the plant material and are unusable and wasted. Another problem is the variation in length of the petioles which results in difficulties in removal of the leaves that are not positioned at the same level (Berg and Deneer, 2012). To overcome this problem, breeders at Rijk Zwaan performed a crossing between a var. *secalinum* and a var. *dulce*, followed by several generations of selection, and developed a new celery cultivar, patented as accession ‘41513’. This cultivar forms not only smaller leaf blades but also very uniform petioles, which minimizes altogether the waste. Although reports describing leaves properties among different celery cultivars are limited, progress was made in understanding the mechanisms underlining the leaf development process. In the aforementioned study of Jia et al. (2015c) leaf development was also studied. The researchers observed that five genes, *AgTCP1*, *AgTCP3*, *AgTCP4*, *AgDELLA*, and *AgARGOS*, involved in cell division, proliferation and differentiation, cytokinin signaling, organ size control, and repression of gibberellic acid signaling, are upregulated during leaf development. The increased expression levels of these genes correlated with the faster growth pattern and increased biomass production characteristic to the ‘Ventura’ cultivar. Jia et al. (2015b) performed a high-throughput sequencing of small RNAs during leaf development in cultivar ‘Ventura’ and identified five microRNAs (miRNAs) that were highly expressed at the four-leaf stage but decreased further on during development. Silencing one or more of these genes or miRNAs can aid the development of celery cultivars with decreased foliage. However this must be done with caution since these miRNAs are expressed in petioles too, albeit at a lower level, and their silencing could therefore negatively impact petiole development. Studies of developmental genes and miRNAs in accessions like ‘41513’ should shed some light on the extent at which the leaf and petiole formation interplay.

### Breeding for Enlarged Hypocotyl Traits

Unlike celery, celeriac has a narrow spread, being concentrated in the north of Europe. The Netherlands, France, Germany, Belgium, and UK are the most prominent countries for celeriac breeding. The main characteristic of celeriac is the development of an enlarged hypocotyl and root that forms a big round structure rich in flavor. The enlarged hypocotyls can be sold on the fresh market or can be processed for the frozen food industry. These applications are also reflected in the breeding goals of this crop.

The roots should be thin and few in number, concentrated on the basal part of the enlarged hypocotyl to prevent damage during the digging for harvesting. Cultivars like ‘Claire’, ‘Diamant’, ‘Camus’, and ‘Rex’ possess a shallower implantation of the roots in the soil, which facilitates the harvesting (Vanparys et al., 2008).

The enlarged hypocotyl of celeriac can grow deep in the soil as well as almost completely above the soil. Cultivars with hypocotyls developing above the soil, like ‘Cesar’, form less roots, which facilitates mechanization and post-harvest cleaning and prevents deformity, but the plants can be more susceptible to frost (Vanparys et al., 2008).

The hypocotyl shape can vary from flat and wide, almost trapezoidal, like in cultivars ‘Balena’ and ‘Elena’, through slightly flat but rounder and higher, like in the case of ‘Alicia’, ‘Cisko’, and ‘Prinz’, to spherical, which is preferred, like the cultivar ‘Rex’. A smooth surface is desired because roughness and grooves increase peel waste. Hypocotyl flesh and skin should be as white as possible throughout the processing; cultivars accumulating anthocyanin are avoided. Browning of the hypocotyls can occur during handling, processing, and storage due to the oxidation of phenolic compounds by polyphenol oxidase (PPO) and peroxidase (POD). Methodology for PPO and POD extraction and quantification from celeriac roots has been established (Aydemir and Akkanli, 2006; Kaiser et al., 2013) and could be used in cultivar development and evaluation. An important characteristic of the internal quality of the flesh is the limited amount of hollowness and sponginess. The cultivars vary in their susceptibility for hollowness from cultivars with few holes, like ‘Cesar’, to cultivars with many holes, like ‘Monarch’.

An important aspect of celeriac breeding is the yield variation between cultivars. Cultivars with a high yield, up to 63 t/ha are ‘Markiz’, ‘Rex’, and ‘Torpedo’, while ‘Cisko’ is low yielding, producing only 41 t/ha. The enlarged hypocotyls can fall in three classes according to their diameter, small (14 cm or less), medium (between 14 and 16 cm), or big (larger than 16 cm). The sorting process determines the use of the crop, either for the fresh market (smaller and medium size) or for the frozen industry (bigger), and can increase the production costs in case of high size variability. The flavor of celeriac hypocotyls is similar to but milder than the flavor of celery leaves and petioles. Phthalides have been isolated from celeriac roots by gas chromatography (GC) (Giuseppe et al., 1996). A comparative study of Abdulmanea et al. (2012) on the flavonoid and isoflavonoid composition of 23 Apiaceae species revealed a different biochanin A and apigenin composition of celeriac roots and leaves. Some flavonoids, like formononetin and kaempferol, are found in hypocotyls but not in leaves.

Although much of the breeding is focused on below the ground traits, celeriac foliage plays an important role in the crop evolution. Cultivars with long petioles and leaves require a longer growing period and therefore an earlier sowing which is economically detrimental. A sufficient amount of green leaves at harvest increases the marketability of fresh celeriac enlarged hypocotyls because it makes them more visually appealing to the consumers (Callens and Vanheule, 2012).

Unfortunately, the efforts to understand the genetic background of important breeding traits of celeriac are limited. Most of the breeding is done *via* crossings, selection, and self-pollination and results in highly homozygous inbred lines. Reports on modern celeriac breeding techniques are scarce. Moravec et al. (1988) unsuccessfully attempted to increase variability in celeriac by polyploidization with colchicine and γ irradiation.

### Intergeneric Breeding

Celery hybridization attempts included intergeneric crossings, such as reciprocal crossings with parsley that yielded intermediate phenotypes (Madjarova and Bubarova, 1978). Late blight resistance introgression has been the objective of the crossings between celery and *A. nodiflorum*, resulting in fertile hybrids (Pink and Innes, 1984) and between celery and *A. chilense*, resulting in low fertility hybrids due to chromosomal rearrangements (Quiros, 1993b). Interspecific crossings between the celery cultivar ‘Tall Utah 52-75’ and *A. prostratum* were employed to introgress leaf miner resistance (Trumble et al., 2000). Ivanova and Bukharov (2009) performed intraspecific crossings between celeriac ‘Cupidon’ and celery ‘Samurai’. The F1 hybrids displayed increased plant height and leaf blade length while for most of the other traits the phenotype was intermediate.

Crossings with wild varieties are very interesting to increase variability, as many wild accessions have unique traits and disease resistances. These crossings sometimes result in reduced petiole quality even after a few generations of backcrossings (Honma and Lacy, 1980). In other cases the desired trait that is introduced from the wild variety is linked to an undesired trait (Navabi et al., 2011). The undesirable traits can be eliminated by genome editing techniques (Schaeffer and Nakata, 2015).

### Breeding for Nutraceuticals and Food Safety

Celery and celeriac are low-calorie vegetables and a reputable medicinal plants, rich in fibers, minerals like calcium, phosphorus, iron, potassium, and magnesium, vitamins like B1, B2, B3, A, and C, and flavonoids (Fazal and Singla, 2012; Sowbhagya, 2014). Slow progression in the development and characterization of cultivars with increased nutrients content is made.

Huang et al. (2016) studied vitamin C levels and its metabolic pathway in the cultivars ‘Liuhe Huangxinqin’ and ‘Ventura’, with the last one containing increased levels of ascorbic acid in both leaves and petioles. The evidence is clear that variation in vitamin C content exists between celery cultivars and screening of gene expression can readily be used in breeding programs.

In an analysis of 11 celery cultivars, Yao et al. (2010) observed a correlation between the phenols composition and antioxidant activity. Shad et al. (2011) compared the nutraceutical composition of three celery cultivars from Pakistan and a wild accession. Variation between the content in different constituents was observed as in the case of the wild *Apium*, which displayed increased content of tannins and phenols. Helay et al. (2015) analyzed the composition of different celeries in selenines, limonene, tannins, phenolic acids, anthocyanins, chlorophyll, carotenoids, and other essential oils. The carotenoids level was the same between cultivars, but the other constituents significantly varied. Wild accessions and local cultivars can potentially improve the ongoing breeding programs by increasing the content of health promoting compounds.

Apigenin is a flavonoid abundantly available in *Apium* leaves. It reportedly has anti-cancer properties [reviewed by Kowalczyk et al. (2017)] and can enhance skeletal muscle hypertrophy and myoblast differentiation in mice (Jang et al., 2017). As aforementioned, the enzyme responsible for conversion of naringenin to apigenin is known and its manipulation leads to an increased level of apigenin (Tan et al., 2017). Yao et al. (2010) concluded that cultivars ‘Shengjie’, ‘27-1-12’, ‘Shandong’, and ‘24-1-6’ possess high apigenin levels.

Marongiu et al. (2013) analyzed the volatile fraction of aerial parts from two wild *Apium* accessions from Portugal and Italy and concluded that they possess strong antifungal activity against *Candida* and *Aspergillus*. The stronger antifungal activity of the Italian accession was attributed to the increase in neophytadiene content. Shad et al. (2011) tested the antibacterial and antifungal activity of the methanolic extract from celery and demonstrated growth inhibition of several bacterial strains. Crossings of these accessions with commercial interesting *Apium* cultivars can potentially lead to the creation of a vegetable with strong antimicrobial and anticancer activities. Such a product could serve as a prophylaxis mean for infection and cancer susceptible groups.

There are potentially toxicological risks for humans associated with celery and celeriac. Furanocoumarins are photoactivated secondary metabolites that are responsible for contact dermatitis of the workers who handle celery plants and are potentially carcinogenic (Fleming, 1990). Petioles and inner leaves contain levels of furanocoumarins in the safe range for human consumption; the higher levels of furanocoumarins are localized mostly in the outer leaves of the plant (Diawara et al., 1995). However, not all coumarins present an implicit health risk, some have pharmacological importance in treating central nervous system diseases (Skalicka-Wozniak et al., 2016).

Celery and celeriac induced allergies are increasing in Central Europe (Andre et al., 1994). They vary from mild manifestation to life-threatening anaphylactic shock (Pauli et al., 1985) and are often associated with sensitization to *Artemisia vulgaris* and *Betula verucosa* pollen, causing the celery-birch syndrome (Lombardero et al., 2004). Four allergens are found in celeriac enlarged hypocotyls but not in leaves, a PR-10 protein (Api g 1) (Breiteneder et al., 1995), a profilin protein (Api g 4) (Scheurer et al., 2000), a flavoprotein (Api g 5) (Bublin et al., 2003), and a non-specific lipid transfer protein 2 (Api g 6) (Vejvar et al., 2013). One allergen, the non-specific lipid transfer protein 1 (Api g 2), is found in celery stalk and aerial parts and undetectable in roots (Gadermaier et al., 2011).

Dolle et al. (2018) studied the allergenic potential of the enlarged hypocotyls of 10 celeriac cultivars. The cultivar ‘Prinz’, that has a high level of ‘Api g 1’ protein, induced the highest reactions in the skin prick test (SPT) and raised the cardiovascular, respiratory, and gastrointestinal symptoms (in patients). Monitoring of the allergen levels in different celery and celeriac cultivars is possible and should be employed in the ongoing breeding programs.

### Breeding for Abiotic Stress and Phytoremediation

Abiotic stress includes drought, heat, and cold, which may increase in particular regions due to climate change, but also salinity, high levels of heavy metals, high light intensity, and UV radiations. All these abiotic stresses induce reactive oxygen species (ROS) accumulation in plants. Plants can withstand some forms of toxicity and many of them can thrive in such austere conditions.

Celery is exceptionally salt tolerant due to the increase in mannitol biosynthesis, which is controlled by mannose-6-phosphate reductase (M6PR) (Gao and Loescher, 2000). *MANNITOL TRANSPORTER 1* (*AgMaT1*), a mannitol transporter protein encoding gene, and two sucrose transporter genes, *SUC UPTAKE TRANSPORT 1* and *2* (*AgSUT1* and *2*), have been described in celery (Noiraud et al., 2000; Noiraud et al., 2001).

Studies in other plant species indicate that metallothioneins (MTs) play a key role in heavy metal and salinity tolerance *via* ROS scavenging (Cobbett and Goldsbrough, 2002; Kumar et al., 2012). These genes can be used as molecular markers for heavy metal tolerant cultivars during the selection phase in breeding programs, or can be engineered in order to obtain new tolerant germplasm. The expression pattern of *AgMT2* was studied in cultivars ‘Liuhe Huangxinqin’, ‘Jinnan Shiqin’, and ‘Ventura’ (Chen et al., 2015). It varied in response to metal ions, heat, drought, cold, and salt stress and was more pronounced in ‘Ventura’.

The expression level of *VIRE2-INTERACTING PROTEIN* (*AgVIP1*) gene was evaluated in three celery cultivars under abiotic stress (Li et al., 2014b). The highest *AgVIP1* level was measured in roots but the gene was also expressed at a lower level in leaves, stems, and flowers in a cultivar dependent manner. These results suggest that *AgVIP1* could be involved in the stress response mechanism of celery and could function as a marker for profiling the unknown resistance status of some celery cultivars since its expression pattern is tightly linked with the genetic background of the cultivars.

The expression profile of the *HEAT SHOCK FACTOR B2* (*AgHSFB2*) gene, which confers protection against heat stress was studied in celery cultivars ‘Liuhe Huangxinqin’, ‘Jinnan Shiqin’, and ‘Ventura’ (Li et al., 2015). *AgHSFB2* upregulation differed between the cultivars, with ‘Ventura’ accumulating faster higher transcript levels as a response to heat. Wu et al. (2018) looked at the expression profile of WRKY transcription factor genes under abiotic stress conditions and observed upregulation of these genes by cold, drought, and salt stress in celery.

Two celery transcription factors, *DEHYDRATION-RESPONSIVE ELEMENT BINDING1* and *2* (*Ag DREB1* and *AgDREB2)* improved cold stress resistance in transgenic *Arabidopsis* (Li et al., 2019) and have potential to be further used for variety screenings in breeding programs.

Through high-throughput screening, Jiang et al. (2014) identified several novel miRNAs targeting abiotic stress genes in the celery cultivar ‘Liuhe Huangxinqin’. In the cultivars ‘Jinnan Shiqin’ and ‘Ventura’, several miRNAs were upregulated by heat and cold stress at cultivar depended rates (Li et al., 2014a). The knowledge of the regulation of stress responses in celery by miRNAs can be used to overexpress or knock-down specific miRNAs and to create new unique genotypes with increased tolerance to abiotic stress.

Several studies regarding the potential of different celery and celeriac cultivars to accumulate heavy metals and pollutants are available. Willaert et al. (1990) evaluated heavy metal accumulation in celeriac and white celery petioles and leaves grown on soils contaminated with heavy metals within the permissible levels. The accumulation of cadmium, manganese, and zinc varied between the investigated cultivars. Twenty-seven Chinese *Apium* accessions were grown in cadmium and lead contaminated soil and the content of heavy metals in the shoots was evaluated; variations between the cultivars were observed, and ‘Shuanggangkangbing’ accumulated the lowest amounts of cadmium and lead (Zhang et al., 2013). The boron uptake in cultivars ‘Emerson Pascal’, an efficient boron user, and ‘S48-54-1’, which is susceptible to low boron, was investigated under low and high boron concentrations (Bellaloui and Brown, 1998). The boron uptake did not differ between the two cultivars but its distribution varied, with ‘Emerson Pascal’ accumulating boron in its shoots and ‘S48-54-1’ in its roots. This indicates that selection of cultivars based on their capacity to accumulate boron in different organs is relevant for preventing the introduction of boron above the toxicity limit in the food chain.

De Temmerman et al. (2012) studied the concentration of arsenic, cadmium, and lead in celeriac plants exposed to atmospheric pollution and observed that the aerial parts of the plant can accumulate all these metals. Arsenic and cadmium can be traced back in the hypocotyl of celeriac plants, unlike lead. These data suggest that *Apium* can be used as a biomarker for atmospheric pollution, since it accumulates more pollutants than other plants. Accumulation of heavy metals into the food chain is however problematic for human health and breeding could help in developing or identifying the cultivars that absorb the least pollutants in the edible parts of the plant (Zhuang et al., 2009).

## Future Perspectives of Celery and Celeriac Breeding

In the future, celery and celeriac breeding will combine the classic approaches that are mostly used today, with new biotechnology advances to speed up the process of marketable cultivar development. The breeding companies opt to invest in new breeding technology based on the importance of the crop, in order to ensure that they will have a turnover from their investment. Given that on the production scale celery and celeriac are considered relatively small crops, these investments are limited, however the growing popularity of them on the markets is promising.

Genomic resources are limited to a few transcriptomic data sets and the whole genome sequence of celery is known and available for Basic Local Alignment Search Tool (BLAST) but not for full download (Feng et al., 2018). The absence of extensive genomic data in celery and celeriac strongly affects the tempo at which new advances in the field of celery research are made. The genomic data will provide a basis for nuclear marker development which are necessary for characterization and selection of material in breeding programs. Detailed gene function studies are also lagging behind in *Apium* and unraveling the mechanisms underlying important breeding traits is crucial. The following quest will be to translate the genomic knowledge into practical breeding by either developing markers for selection of pre-breeding material or by engineering specific traits in existing cultivars.

In the near future, celery and celeriac breeding will be focused almost exclusively on hybrid cultivars development. F1 hybrids are increasingly interesting in the context of increasing crop productivity for overall reduction of the environmental impact of agriculture. OP varieties will be produced at a limited extent mostly for markets that have a strong tradition around these cultivars (an example is the popularity of ‘Tango’ cultivar on USA markets). OP varieties are however not desired by most markets because of their variability in extreme conditions.

A prominent problem in the current *Apium* breeding is the lack of stable CMS lines, which still complicates hybrid seed production. Besides the well-known CMS introgression *via* asymmetric protoplasts fusion or sexual crossings with distantly related species, researchers should focus on better understanding the mechanisms behind mitochondrial involvement in this trait. The majority of the molecular markers developed in celery are nuclear. Cytoplasmic markers, both of chloroplast and mitochondrial origins, are essential for characterization of plant material resulting from protoplast fusions and sexual crosses that aim to develop CMS lines. The *Daucus carota* mitochondrial plastid (*Dc*MP) sequence is a carrot plastid genome sequence which is polymorphic among different Apiaceae species and gives a PCR product of 1,618 nt in carrot, 500 nt in celery, and 400 nt in coriander (*Coriandrum sativum*) (Iorizzo et al., 2012) and can be used to discriminate celery and carrot chloroplasts. No mitochondrial specific markers have yet been reported in celery.

In current breeding practice, molecular markers like single-nucleotide polymorphism (SNPs) are used for specific traits like disease resistance (*Fusarium*, *Septoria*) in mapping populations. In the future, the research groups working on celery molecular markers will probably focus on establishing SNP markers for most of the important traits and cytoplasmic markers. Doubled haploid technology facilitates the development of homozygous lines that have the potential to be used in cultivar development, F1 hybrid production, or reverse breeding. Haploid plants have been obtained in different species from microspore or anther cultures and lately, by engineering the centromere-specific histone 3 (CENH3) variant. Research on haploid production by CENH3 engineering or microspore and anther culture has been published for carrot (Gorecka et al., 2009; Li et al., 2013; Dunemann et al., 2014) but not yet for celery.

Development of cultivars with increased tolerance to biotic stress must be a continuous breeding goal in celery, because the pathogen effectors evolve to suppress eventually the plant innate immune response. In the current agriculture practice the emphasis is on reducing pesticide sprayings and on organic practices, which will benefit significantly from the disease resistant cultivars.

High temperatures and prolonged drought can have a big toll on the yield of *Apium* since it thrives best in relatively cool weather (16–21°C) and requires irrigation (Malhotra, 2006). Breeders and scientists will have to address the climate change associated problems and focus on developing celery and celeriac cultivars that can withstand extreme environmental conditions. With a classic breeding cycle of 8 to 15 years, there might not be much time left. However, alternative breeding techniques, like genome editing by CRISPR-Cas9 could be employed to speed up the process.

**Figure 2:**
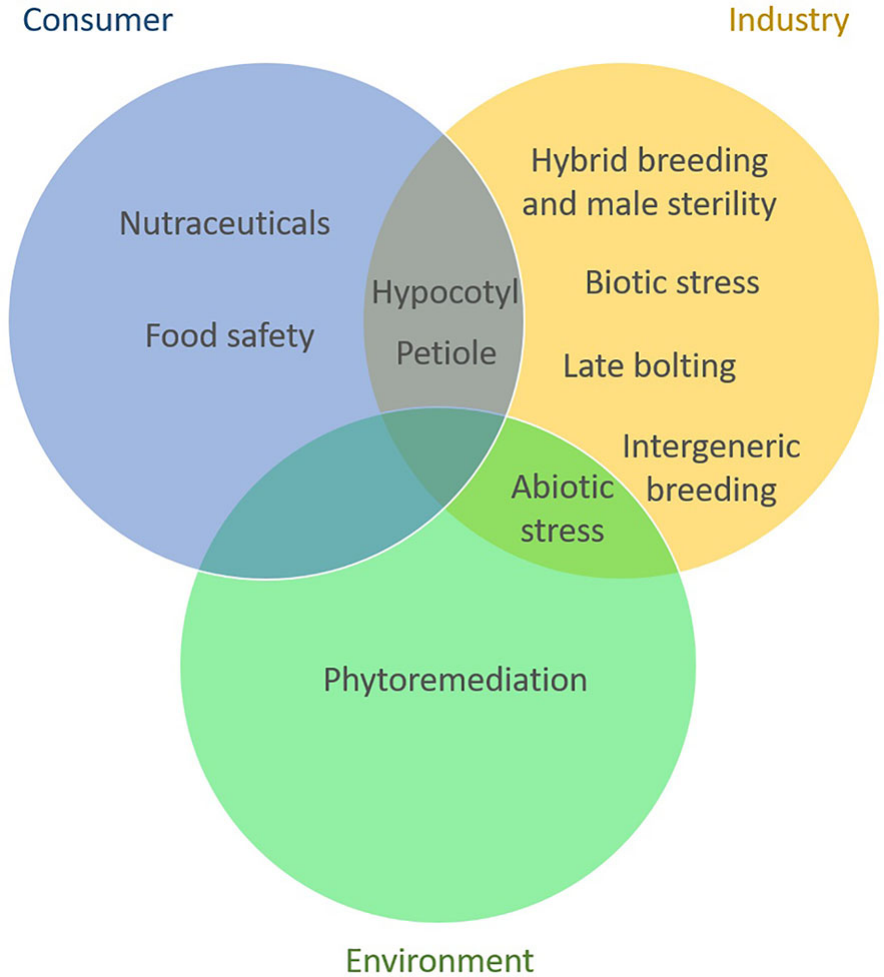
*Apium* breeding objectives per stakeholder. Most of the current breeding is focused on traits that are mainly relevant for growers, breeders, and the processing sector. The interest in breeding for consumer related traits is increasing and some of these traits are also beneficial for the industry. Goals related to sustainability and environment are currently least tackled in celery and celeriac breeding.

Adopting breeding goals that are more consumer oriented has the potential to promote a positive image of breeding and biotechnology. Development of cultivars with increased content in health promoting compounds, with pleasant sensorial characteristics and decreased allergens content can offer new perspectives in breeding celery and celeriac. We depicted in **Figure 2** the distribution of celery breeding goals per beneficiary sector.

## Author Contributions

Review conception and design: SB, HC, JH, TE, DG. Literature study: SB. Drafting of manuscript: SB, HC, JH, TE, DG.

## Conflict of Interest

The authors declare that the research was conducted in the absence of any commercial or financial relationships that could be construed as a potential conflict of interest.
